# Level of Amyloid-β (Aβ) Binding Leading to Differential Effects on Resting State Functional Connectivity in Major Brain Networks

**DOI:** 10.3390/biomedicines10092321

**Published:** 2022-09-19

**Authors:** Eva Y. W. Cheung, Anson C. M. Chau, Yat-Fung Shea, Patrick K. C. Chiu, Joseph S. K. Kwan, Henry K. F. Mak

**Affiliations:** 1 Department of Diagnostic Radiology, LKS Faculty of Medicine, The University of Hong Kong, Hong Kong; 2 School of Medical Health and Sciences, Tung Wah College, 19/F, 31 Wylie Road, Ho Man Tin, Hong Kong; 3 Medical Radiation Science, Allied Health and Human Performance Unit, University of South Australia, City East Campus, Bonython Jubilee Building, 1-26, Adelaide, SA 5001, Australia; 4 Division of Geriatrics, Department of Medicine, Queen Mary Hospital, Hong Kong; 5 Department of Brain Sciences, Imperial College London, London W12 0NN, UK; 6 State Key Laboratory of Brain and Cognitive Sciences, The University of Hong Kong, Hong Kong; 7 Alzheimer’s Disease Research Network, The University of Hong Kong, Hong Kong

**Keywords:** Alzheimer’s disease, mild cognitive impairment, vascular dementia, Amyloid-β protein (Aβ), resting state functional MRI (rsMRI), 18F-flutemetamol, default mode network (DMN), central executive network (CEN), salience network (SN), self-referential network (SRN), sensory motor network (SMN)

## Abstract

Introduction: Amyloid-β protein (Aβ) is one of the biomarkers for Alzheimer’s disease (AD). The recent application of interhemispheric functional connectivity (IFC) in resting-state fMRI has been used as a non-invasive diagnostic tool for early dementia. In this study, we focused on the level of Aβ accumulated and its effects on the major functional networks, including default mode network (DMN), central executive network (CEN), salience network (SN), self-referential network (SRN) and sensory motor network (SMN). Methods: 58 participants (27 Hi Aβ (HiAmy) and 31 low Aβ (LowAmy)) and 25 healthy controls (HC) were recruited. [^18^F]flutemetamol PET/CT was performed for diseased groups, and MRI scanning was done for all participants. Voxel-by-voxel correlation analysis was done for both groups in all networks. Results: In HiAmy, IFC was reduced in all networks except SN. A negative correlation in DMN, CEN, SRN and SMN suggests high Aβ related to IFC reduction; However, a positive correlation in SN suggests high Aβ related to an increase in IFC. In LowAmy, IFC increased in CEN, SMN, SN and SRN. Positive correlation in all major brain networks. Conclusion: The level of Aβ accumulated demonstrated differential effects on IFC in various brain networks. As the treatment to reduce Aβ plaque deposition is available in the market, it may be an option for the HiAmy group to improve their IFC in major brain networks.

## 1. Introduction

Amyloid-β protein (Aβ) deposition is one of the signature biomarkers for Alzheimer’s disease (AD). Since the 2000s, the in-vivo detection of Aβ pathology became feasible in the human brain via the usage of C-11 and F-18 labeled radiopharmaceuticals; their chemical features allowed binding to Aβ as ligands, and the radioactive labels can be visible in the positron emission tomography (PET) imaging. Pittsburg compound B was labeled with C-11 and acted as an Aβ tracer to indicate the location of Aβ deposition in patients with AD in PET imaging [1]. A recent study used [^18^F]flutemetamol as radiopharmaceutical and showed that the location of high concentration of Aβ protein observed in PET images had high plaque density in the brain regions, which were consistent with the support of post-mortem results [2].

Neuropathological staging in AD based on Aβ protein deposition level has been suggested, which indicates that Aβ deposited follows the pattern from frontal, parietal, temporal and occipital throughout the neocortex [3]. However, the development of AD is complex. Simply knowing the route of Aβ protein deposition is not sufficient in the prediction of the disease progression. Other biomarkers, such as cardiovascular risks and brain connectivity, may help to improve our understanding of the future decline to supplement the Aβ accumulation. Interhemispheric functional connectivity (IFC) is one of the biomarkers that dementia patients demonstrate impairment; it has long been discussed and was measured by electroencephalogram (EEG) [4]. However, the EEG signal can easily be interfered with by other physical connectivity and was not ideal in the application for early diagnosis. Recently, researchers employed resting-state functional MRI (rs-FMRI), to study IFC. Voxel mirror homotopic connectivity (VMHC) is a method to analyze IFC in a voxel-by-voxel manner with high accuracy and precision; it has been employed in previous studies to differentiate AD, mild cognitive impairment (MCI) from healthy control, based on the aberrant connectivity in different brain regions [5,6,7] which is sensitive to the early functional change of the brain in dementia patients.

A previous study showed that Aβ protein starts to accumulate in the brain regions in the default mode network (DMN) and central executive network (CEN) [8]. Some researchers suggested that the accumulated Aβ reduced brain functional connectivity [8], while some demonstrated that it increased functional connectivity [9,10]. The difference in functional connectivity change may be affected by the level of Aβ protein accumulated as well as the regions of deposition; it is worthwhile to investigate how the Aβ protein deposition correlates with IFC, and whether the level of Aβ protein deposition may have different effects on brain functional connectivity in different brain networks. In this study, we aimed to evaluate the association between regional Aβ protein accumulation with IFC, in view of different brain functional networks, which may improve our understanding of the effects of Aβ protein accumulation on different brain networks.

## 2. Method

### 2.1. Participants

From June 2017 to June 2019, participants who were diagnosed with AD, MCI or vascular dementia (VD), aged over 55 years old and have an informant were recruited consecutively from the memory clinic of a university hospital. Participants being diagnosed with cancer within 5 years, psychiatric illness; had a history of regular drug or alcohol abuse, migraine, head injury, seizure, and stroke; being diagnosed with active infection and organ failure recently; had physical barriers such as deafness were excluded from the study. Informed consent was obtained from all participants and from the caregivers of demented participants. Institutional Review Board (IRB) approval was obtained for the research protocol.

All participants underwent clinical evaluation, neuropsychological test (the Hong Kong version of Montreal Cognitive Assessment (HK-MoCA) [11], [^18^F]flutemetamol PET/CT scan, magnetic resonance imaging (T1 MPRAGE, rs-fMRI, ASL perfusion, MR angiography). All imaging scans were conducted within one week. A group of HC was recruited from the university hospital, with age-matched with all recruited participants (diseased group). HC underwent clinical evaluation, neuropsychological test and MRI only, due to the restricted application of radiopharmaceuticals to HC.

### 2.2. Dementia/Cognitive Impaired Subtype Classification

The final diagnosis of each participant was determined by a multi-disciplinary panel, which consisted of two geriatricians (SYF and CPKC) and one neuroradiologist (MHKF) based on the imaging (MRI, [^18^F]flutemetamol PET/CT scan), clinical (baseline and follow-up) and neuropsychological (HK-MoCA test) findings. The clinical diagnostic criteria for AD and MCI patients were in reference to the McKhann et al., 2011 study [12] in addition to a positive [^18^F]flutemetamol PET/CT scan result. For VD patients, the diagnosis was made with reference to the clinical criteria described in the Roman 1993 study [13], a negative [^18^F]flutemetamol PET/CT scan, as well as macro-vascular MRA and microvascular MRI abnormalities. The MR ASL perfusion scan provided additional information related to the perfusion patterns when it was necessary.

## 3. Imaging Protocol

### 3.1. [^18^F]Flutemetamol PET/CT Image Acquisition

The Aβ protein accumulation was assessed by [^18^F]flutemetamol PET/CT image. All participants were instructed to fast for 6 h before the tracer injection. A dosage of 5mCi of [^18^F]flutemetamol was injected intravenously into the participant within 40 s. The participant had to wait 90 min in a dimmed room for the tracer uptake before scanning. 3D mode PET/CT scanner was used, with a slice thickness of 2 mm, pixel size of 2 mm, and matrix size of 128 × 128. A post-smoothing filter of full-width half-maximum (FWHM) of 5 mm was applied. Scanning time was limited to 30 min.

### 3.2. MRI Acquisition: T1 MPRAGE Images

MRI images were acquired using a Philips Achieva 3T equipped with a 32-channel phased-array head coil. A T1 MPRAGE (magnetization prepared rapid acquisition gradient-echo): repetition time [TR] = 6.75 ms, echo time [TE] = 3.163 ms, inversion time [TI] = 844 ms, flip angle = 8°, slice thickness = 1.2 mm, 256 sagittal slices; acquisition matrix = 256 × 256, field of view = 256 × 256 mm^2^, voxel size = 1 × 1 × 1.2 mm^3^, bandwidth = 241 Hz/pixel was obtained.

### 3.3. MRI Acquisition: Resting State Functional Images

Each participant was asked to relax and remain quiet during the scan, with their eyes kept closed but not fall asleep. Resting state functional MRI (rs-fMRI) images were obtained using multi-echo echo planar imaging (EPI) sequence, with following parameters: 180 time points; TR = 2000 ms, TE = 30 ms, Flip angle = 90°, slice thickness = 4 mm, FOV = 230 × 230 mm^2^, acquisition matrix = 72 × 72, 36 slices, pixel dimension: 144 × 144 mm^2^, voxel size = 1.6 × 1.6 × 4 mm^3^).

## 4. Preprocessing of Images

### 4.1. Pre-Processing of Resting State Functional MRI

Data pre-processing was conducted by Data Processing Assistant for Resting State fMRI (DPARSF) v.4.4 (http://www.rfmri.org/dparsf, accessed on 22 June 2022); it is a software built on the MATLAB platform 2018a (The MathWorks, Inc., Natick, MA, USA), with Statistical Parametric Mapping software (SPM12 http://www.fil.ion.ucl.ac.uk/spm/software/spm12/, accessed on 22 June 2022). Pre-processing was performed with standard processing steps as follows [14]: 180 time points were collected for each participant. The first 10 image volumes were removed to eliminate magnetic saturation effects. The remaining 170 images were re-sliced for motion and time correction with reference to the 35th slice. A maximum of 2 mm displacement in any direction (x, y, and z) and a maximum 2-degree rotation in any direction (x, y, and z) during the rs-fMRI scan was limited. To normalize the fMRI image spatially, the high-resolution T1-weighted images were co-registered to the mean fMRI in each participant; the resulting aligned images were segmented and transformed into Montreal Neurological Institute (MNI) space using the DARTEL toolbox to generate a group template. Then, the motion-corrected fMRI were specifically normalized to the group template using the transfer parameter estimated through DARTEL segmentation and resampled to a voxel size of 3 × 3 × 3 mm. The fMRI images were smoothed using 4 × 4 × 4 mm full width half maximum (FWHM) Gaussian kernel. To minimize physiological noise from low-frequency drift and high-frequency, temporal 0.01–0.1 Hz band-pass filtering was applied. 0.5 frame-wise displacement (FD) threshold was applied to minimize the motion artefacts, and the bad points were interpolated using the nearest neighbor algorithm. Finally, a nuisance linear regression was performed at one time point earlier, using the global signal, six head motion parameters, the white matter and the cerebrospinal fluid, as well as the 12 corresponding squared items (Friston 24-parameter model) as covariates.

### 4.2. Voxel-Mirrored Homotopic Connectivity VMHC

To analyse interhemispheric functional connectivity (IFC) on the rs-fMRI, voxel-mirrored homotopic connectivity (VMHC) was employed. The pre-processed images were transformed into a symmetric brain template, which was obtained by flipping the right or left hemispheres along the X-axis midline and took an average with the original image. The T1w and rs-fMRI images were normalized to the MNI space in each participant, and then co-registered to the group-specific symmetric template. The IFC was analysed with reference to the Pearson correlation between exact paired voxels located at the same location but in different hemispheres. The resultant correlation coefficients were used to compose the VMHC map. The VMHC value of a particular region of interest (ROI) can be extracted from the corresponding VMHC map for further analysis. All the results were presented using the unilateral (left side only) brain mask, due to the fact that VMHC values were symmetric [15].

### 4.3. Total Intra-Cranial Volume and Gray Matter Volume Calculation

The total intracranial volume and gray matter volume were calculated using the FreeSurfer software package (http://surfer.nmr.mgh.harvard.edu/ version 7.1, accessed on 22 June 2022) [16]. The subcortical volume segmentation and measurement of different brain structures were done automatically with the procedures listed as follows [17]: Briefly, the process includes motion correction, removal of non-brain tissue using a hybrid watershed/surface deformation procedure [18] automated Talairach transformation, segmentation of the subcortical white matter and deep gray matter volumetric structures (including the putamen, amygdala, caudate, hippocampus, and the ventricles) [17,18] intensity normalization, tessellation of the gray-white matter boundary, automated topology correction [19,20] and surface deformation following intensity gradients to optimally place the gray matter/CSF borders and gray-white matter at the location where the greatest shift in intensity defines the transition to the other tissue class [21,22,23].

## 5. Image and Statistical Analysis

### 5.1. Participants and Demographics

Kruskal Wallis one-way analysis of variance (ANOVA) test was performed to assess the significant difference in demographic data between the three groups (HiAmy, LowAmy and HC) using SPSS version 25.0 (SPSS Inc, Chicago, IL, USA).

### 5.2. Global Aβ Protein Accumulation Analysis: PET/CT Images

The global SUV of each participant was obtained from their individual PET image; it is the average SUV of the whole brain. The mean global SUVr was calculated as following,

$$\mathrm{SUVr}=\frac{\mathrm{SUV}\;\mathrm{of}\;\mathrm{the}\;\mathrm{whole}\;\mathrm{brain}}{\mathrm{mean}\;\mathrm{SUV}\;\mathrm{of}\;\mathrm{pons}}$$

### 5.3. Regional Aβ Protein Accumulation Analysis

PET images were normalized to custom template space (European scaled MNI spaced) using the normalization parameters of their co-registered MRI scans, as computed with SPM12 unified segmentation. The mean SUV map for each group was computed to allow direct assessment of the pattern within the group. SUV of pons of each group was calculated as a reference for SUVr calculation. SUVr was computed based on the following equation:

$$\mathrm{SUVr}=\frac{\mathrm{SUV}\;\mathrm{of}\;\mathrm{that}\;\mathrm{voxel}}{\mathrm{mean}\;\mathrm{SUV}\;\mathrm{of}\;\mathrm{pons}\;\mathrm{of}\;\mathrm{the}\;\mathrm{group}}$$

The mean SUVr map of each group was obtained. The [^18^F]flutemetamol uptake in white matter was non-specific, i.e., the uptake in the white matter region was not specifically related to Aβ. Therefore, a white matter mask was created using WFU Pickatlas version 3.0.5 [24] and applied on the SUVr maps, to remove the SUVr from white matter with non-specific uptake of [^18^F]flutemetamol.

### 5.4. IFC Analysis: VMHC Map Gerenrated from fMRI

IFC analysis was based on the VMHC values, which were extracted from different brain regions on the VMHC map in a voxel-wise manner. A one-sample independent sample *t*-test was conducted to assess the IFC based on VMHC maps within the same group using DPABI software. A two-sample *t*-test was performed to assess the VMHC maps between the diseased group and HC, with age, sex and total intracranial volume as covariates. In the VMHC map, the minimum cluster size was set to 100 voxels. The significance level of the group difference was threshold based on the two-tailed Gaussian Random Field (GRF) theory to correct for multiple comparison, with a voxel level of *p* < 0.01 and cluster level of *p* < 0.05 [14].

### 5.5. Correlation Analysis between Aβ Protein Accumulation and IFC

VMHC and SUVr maps were used to conduct a voxel-wise correlation analysis using the DPABI v 4.0. Pearson’s correlation coefficient (r) was calculated to assess the relationship between the Aβ protein accumulation and IFC. The voxel-wise correlation coefficient was computed and displayed as correlation maps, with total intra-cranial volume, age, sex as covariates.

The brain regions with statistically significant correlation (*p* < 0.05) were presented in correlation maps with respect to a collection of brain networks, which were identified by Mantini and his colleagues based on BOLD signals [25] and electroencephalography [26]; they are default mode network (DMN), central executive network (CEN), salience network (SN), self-referential network (SRN) and sensory motor network (SMN); these networks have been employed as brain templates in fMRI studies [27,28].

## 6. Results

### 6.1. Demographics

A total of 58 patients and 25 healthy controls were recruited in this study. There were 16 patients with AD, 11 patients with MCI with positive amyloid scan (AmyMCI), 18 patients with MCI with negative amyloid scan (NamyMCI) and 13 patients with VD. With respect to the SUVr threshold established by Thurfjell and his colleagues for differentiating positive and negative scan in global binding [29], the global SUVr of 0.62 was used to classify the Low and High amyloid group. In this study, all participants with global SUVr less than 0.62 was classified as low amyloid (LowAmy) group, those with SUVr higher than 0.62 was classified as high amyloid (HiAmy) group. As a result, there were 27 and 31 participants was classified in HiAmy (AD and AmyMCI) group and in LowAmy (NamyMCI and VD) group respectively.

Nonparametric Kruskal–Wallis ANOVA test showed no significant differences in age and sex between the three groups, nor in the neuropsychological test between the two groups. Results are listed in Table 1.

### 6.2. Aβ Protein Accumulation (SUVr) Maps

**Figure 1:**
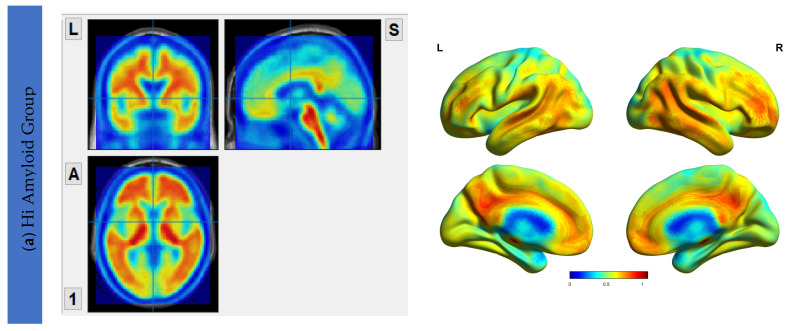

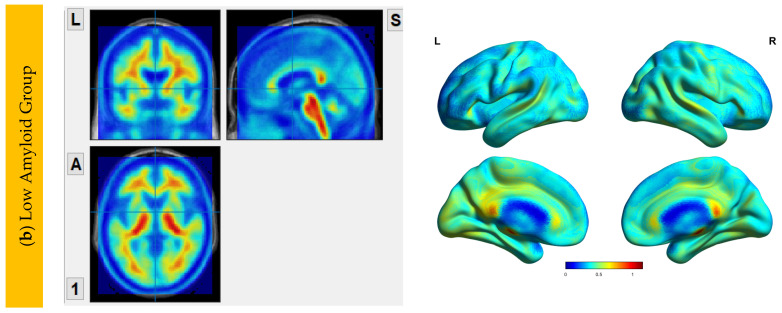
[^18^F]flutemetamol PET/CT imaging showing Aβ protein accumulation (SUVr) Maps, showing regions of Aβ accumulations from voxel-wise analysis. (**a**) shows the regions where Aβ protein distribution in the HiAmy group. (**b**) shows the regions where Aβ protein distribution in the LowAmy group. The voxel-wise map is adjusted for age and sex. The blue and red colours illustrate the SUVr values according to the scale on the right. When compare Figure 1a,b, the Aβ protein accumulation was much higher in the HiAmy group when compared to the LowAmy group. HiAmy: Patient group with global SUVr > 0.62. LowAmy: Patient group with global SUVr < 0.62.

The Aβ protein accumulation was assessed using [^18^F]flutemetamol uptake maps. The maps for HiAmy and LowAmy groups were displayed in Figure 1. SUVr is a ratio from 0 to 1, with 0 indicating no Aβ protein accumulation and 1 indicating high Aβ protein accumulation.

### 6.3. Interhemispheric Functional Connectivity (VMHC) Maps

**Figure 2:**
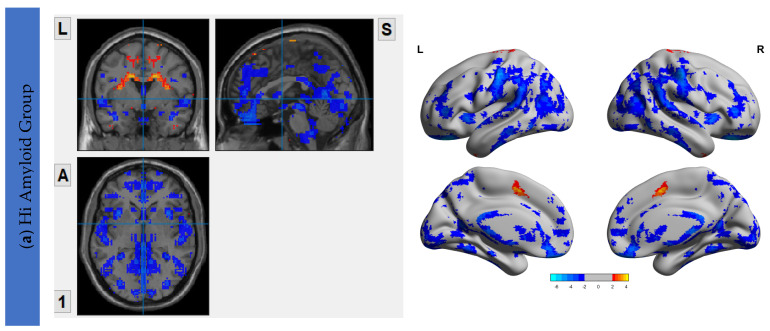

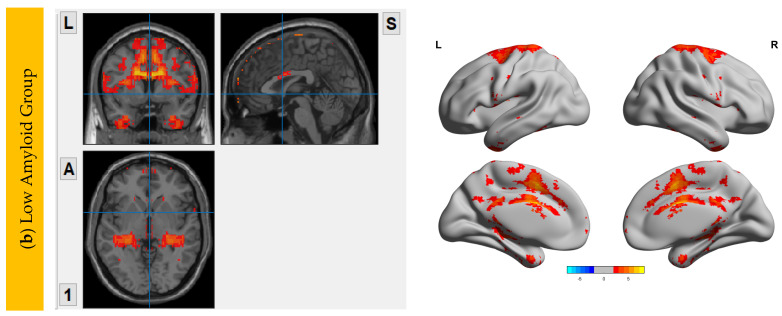
VMHC Maps showing interhemispheric functional connectivity (IFC) of HiAmy and LowAmy groups when compared to HC. (**a**) showed Voxel-wise two-sample *t*-tests of VMHC maps from HiAmy when compared to HC. (**b)** showed Voxel-wise two-sample *t*-tests of VMHC maps from LowAmy when compared to HC. All comparisons were adjusted for age, sex, and total intracranial volume, with Gaussian random field correction. The significance threshold at the voxel level was set at *p* < 0.01; and at cluster level was set at *p* < 0.05. The blue and red colors illustrate significant t values according to the scale on the right. VMHC: Voxel Mirrored Homotopic Connectivity. HC: Health Control. HiAmy: Patient group with global SUVr > 0.62. LowAmy: Patient group with global SUVc < 0.62.

The VMHC maps for the HiAmy and LowAmy groups when compared to HC were showed in Figure 2. For the HiAmy group, the VMHC maps showed reduced functional connectivity in most regions in DMN, CEN, SRN and SMN, except in regions of salience network, including anterior insular, anterior cingulate gyrus, putamen, pallidum, caudate and amygdala. While for LowAmy group, the VMHC maps showed increased functional connectivity in the precentral gyrus, superior frontal gyrus, inferior frontal operculum, rolandic operculum, supplementary motor area, anterior insular, middle cingulate and caudate.

## 7. Correlation Analysis between Aβ Accumulation and IFC

### 7.1. Within DMN

**Figure 3:**
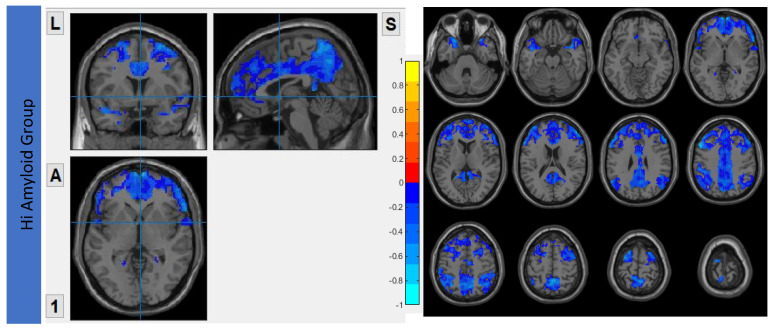

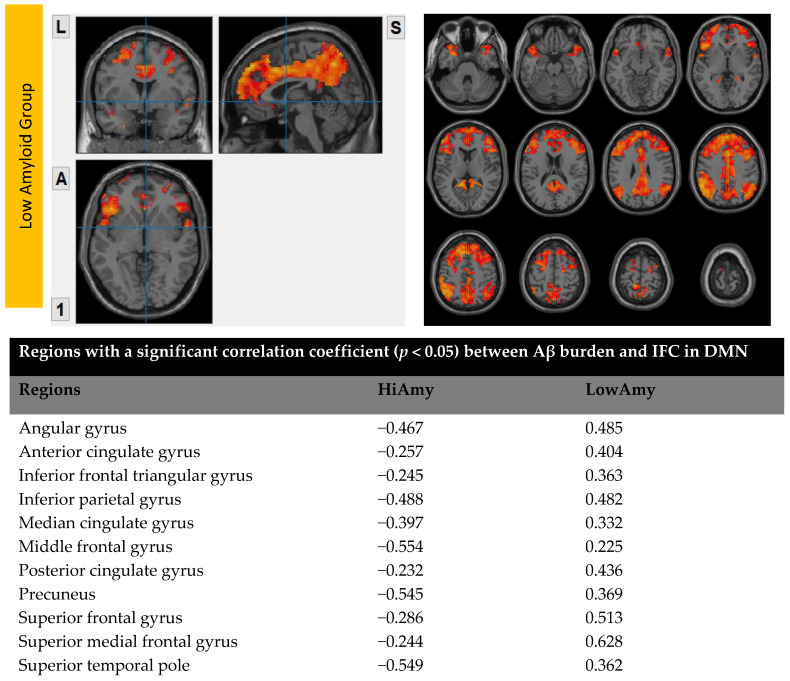
Correlation between Aβ burden and IFC in DMN.

In the HiAmy group, voxel-wise correlation analysis showed negative correlation (*r* = −0.232 to −0.554, *p* < 0.05) in the correlation map, indicating the Aβ accumulation relates to the reduction of functional connectivity of that region. However, in the LowAmy group, voxel-wise correlation analysis showed positive correlation (*r* = 0.225 to 0.628, *p* < 0.05) in the correlation map, indicating the Aβ accumulation relates to the increased functional connectivity of that region. Details are shown in Figure 3.

### 7.2. Within CEN

**Figure 4:**
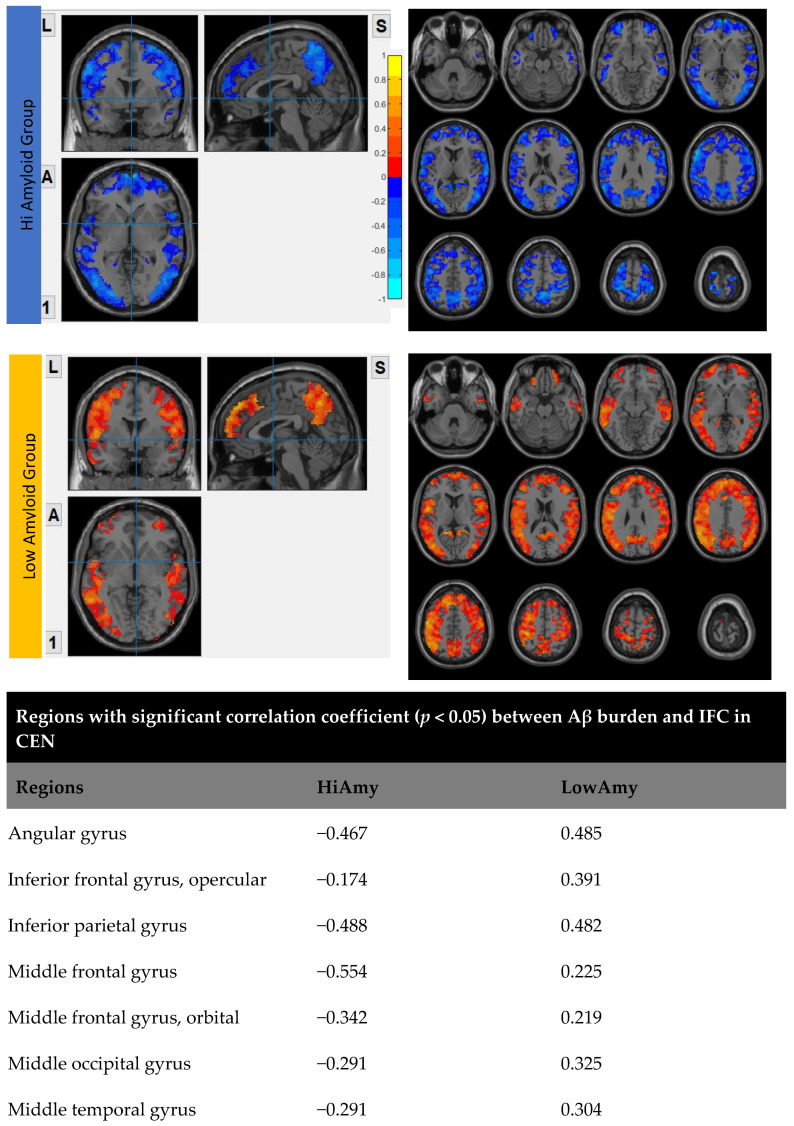

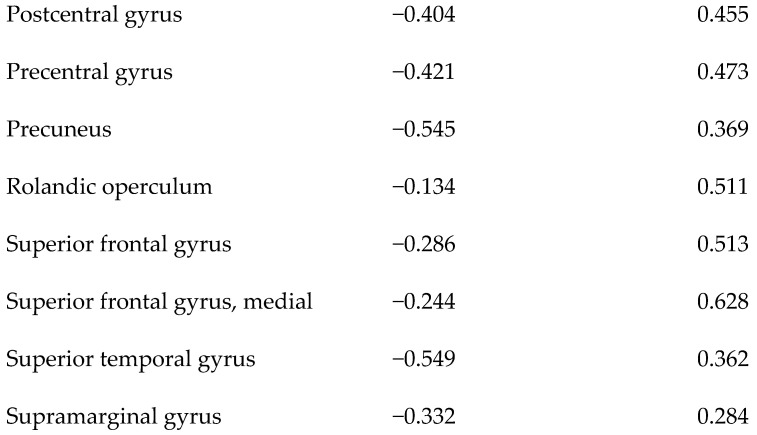
Correlation between Aβ burden and IFC in CEN.

In the HiAmy group, voxel-wise correlation analysis showed negative correlation (*r* = −0.134 to −0.545, *p* < 0.05) in the correlation map, indicating the Aβ accumulation relates to the reduction of functional connectivity of that region. However, in the LowAmy group, voxel-wise correlation analysis showed positive correlation *(r* = 0.225 to 0.628, *p* < 0.05) in the correlation map, indicating the Aβ accumulation relates to the increased functional connectivity of that region. Details were shown in Figure 4.

### 7.3. Within SN

**Figure 5:**
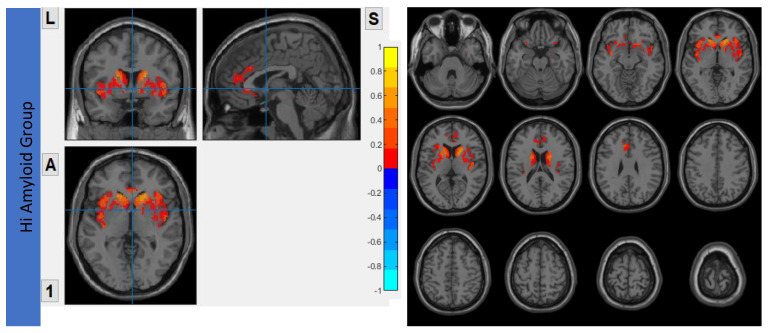

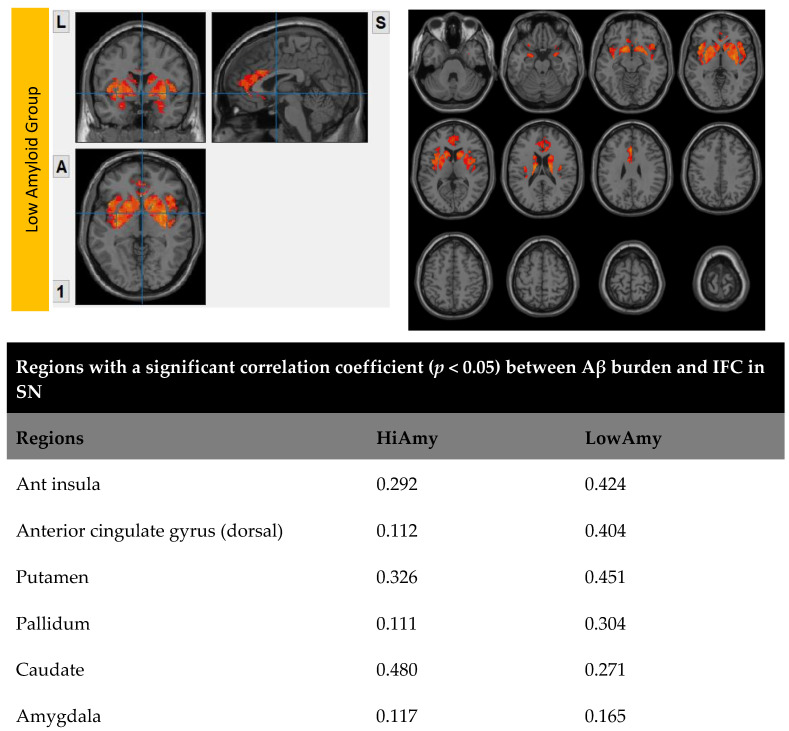
Correlation between Aβ burden and IFC in SN.

Both HiAmy and LowAmy groups showed a positive correlation in the correlation map, ranges from *r* = 0.111 to 0.480, *p* < 0.05 and 0.165 to 0.451, *p* < 0.05 respectively, indicating the Aβ accumulation relates to the reduction of functional connectivity of that region. Details were shown in Figure 5.

### 7.4. Within SRN

**Figure 6:**
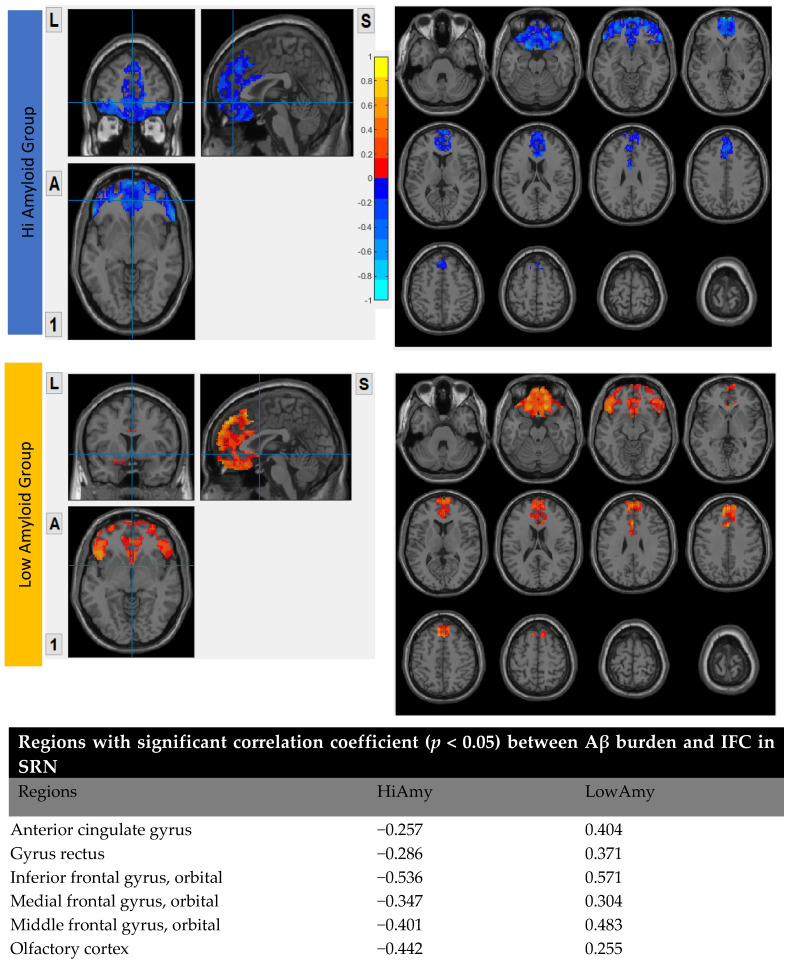

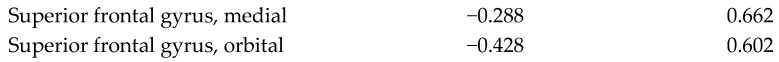
Correlation between Aβ burden and IFC in SRN.

In the HiAmy group, voxel-wise correlation analysis showed negative correlation (*r* = −0.257 to −0.536, *p* < 0.05) in the correlation map, indicating the Aβ accumulation relates to the reduction of functional connectivity of that region. However, in the LowAmy group, voxel-wise correlation analysis showed positive correlation *(r* = 0.255 to 0.662, *p* < 0.05) in the correlation map, indicating the Aβ accumulation relates to the increased functional connectivity of that region. Details were shown in Figure 6.

### 7.5. Within SMN

**Figure 7:**
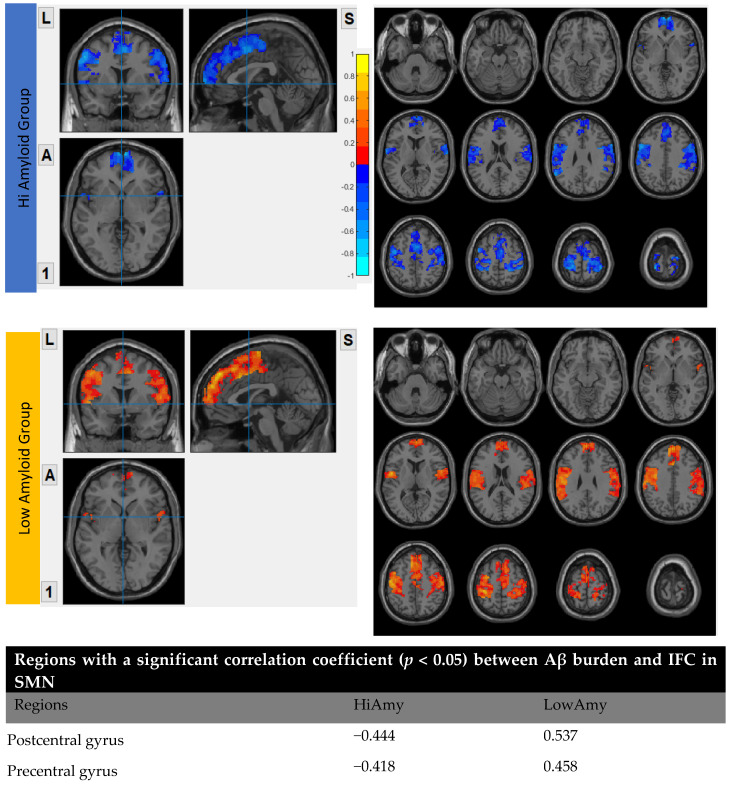

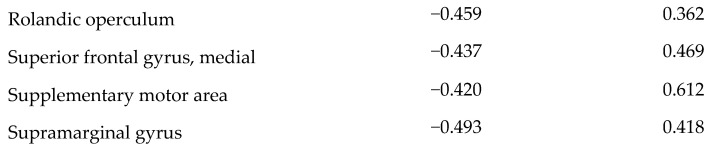
Correlation between Aβ burden and IFC in SMN.

In the HiAmy group, voxel-wise correlation analysis showed a negative correlation (*r* = −0.418 to −0.493, *p* < 0.05) in the correlation map, indicating the Aβ accumulation relates to the reduction of functional connectivity of that region. However, in the LowAmy group, voxel-wise correlation analysis showed positive correlation *(r* = 0.362 *to* 0.612, *p* < 0.05) in the correlation map, indicating the Aβ accumulation relates to the increased functional connectivity of that region. Details were shown In Figure 7.

## 8. Discussion

The findings of this study demonstrated that the level of amyloid-β accumulated have differential effects on IFC in different brain networks. For the HiAmy group (global SUVr > 0.62), the regional Aβ deposition associated with reduction in IFC in DMN, CEN, SRN and SMN; whereas it was associated with increased IFC in SN only. For the LowAmy group (global SUVr < 0.62), the regional Aβ deposition was associated with increased IFC in all important brain networks.

Brain networks can be defined based on structural connectivity and functional connectivity, which is measured by diffusion tensor imaging (DTI) or functional MRI (fMRI), respectively [30]; they have been characterized by brain regions of a group and the connections between them [31]. People developed impairments in association with dysfunction in major functional networks. AD patients performed poorly in tasks that require interhemispheric brain functions [32]. Thus, the interhemispheric FC has been employed as early diagnosis of AD, MCI [5,32,33,34] and vascular dementia [7], based on the aberrant functional connectivity pattern from healthy controls. IFC reduction relates to the decline of regional brain function; whereas IFC indicate the activated function of that brain region. The pattern of aberrant IFC indicated deficits of function with reference to the brain regions.

Alzheimer’s disease pathogenesis is widely believed to be driven by the Aβ deposition [35]. Braak and Braak 1997 demonstrated that Aβ accumulated from the frontal lobe of the brain (stage A), to the intermediate stage (stage B) at the olfactory, medial frontal orbital gyrus, inferior frontal operculum, inferior frontal orbital gyrus, calcarine, lingual gyrus and cuneus; to the late stage (stage C) including precuneus and superior medial frontal gyrus [36]. AD patients developed substantial Aβ deposition from a decade, and its pattern of deposition was not directly reflecting the deficits of dementia patients. The deposition may have differential effects on the IFC pattern in the brain functional network.

In view of the triple network model, the major functional networks are DMN, CEN and SN. DMN is the first to be identified. Within functional imaging studies, hippocampus, posterior cingulate gyrus and angular gyrus are associated with autobiographical memory and episodic memory retrieval. The medial prefrontal cortex is associated with self-related and social cognitive processes, which governed value-based behavior and emotion regulation. In this study, the HiAmy group were AD and MCI patients with global SUVr higher than 0.62, their PET images showed that the high Aβ deposition cover olfactory, medial frontal orbital gyrus, inferior frontal operculum, inferior frontal orbital gyrus, calcarine, lingual gyrus and cuneus (stage B) and precuneus, superior medial frontal gyrus (stage C) regions [36]. The negative correlation that Aβ deposition in DMN is associated with reduced IFC. Episodic and autobiographical memory loss are the classical symptoms of AD, the connectivity of both the posterior cingulate gyrus and hippocampus have been implicated in memory deficits in AD [37,38]. Our result was consistent with the result of Hampton et al. 2020, they concluded that high Aβ deposition group had an effect of lower connectivity in DMN, and it increased longitudinal atrophy within DMN [39]. With respect to the MCI, amyloid positive MCI (HiAmy group) had more widespread reduction of DMN connectivity than those with amyloid negative scan [40]. Our result showed that Aβ deposition is low in most of the brain regions in the LowAmy group. The positive correlation between IFC and Aβ deposition indicated that a low level of Aβ deposition promote the IFC in DMN. Similar results has been obtain in Cai et al. 2015 study, where they concluded that increased FC indicated MCI patients recruited other brain resources to compensate for the loss of cognitive function [41].

CEN is an important network for active maintain and manipulate information in working memory, for problem-solving and decision-making with reference to goal-directed behavior [42,43]. Previous studies suggested that CEN disruption is widespread in AD patient [44,45]. Connectivity deficits were found in the dorsal lateral prefrontal cortex (dlPFC) and posterior prefrontal cortex (PPC) and also on fronto-parietal regions. Deficits in CEN can be resulted due to reduced connectivity between nodes in CEN [46]. Similar to the DMN region, the HiAmy group in this study had high Aβ deposition in CEN, which correlated negatively with IFC. Our result was coherent with Mormino et al. 2011 study, which showed decreased FC in medial prefrontal cortex and angular gyrus (CEN regions) with high Aβ deposition in AD [47]. However, low Aβ deposition was found at the dorsal and anterior medial prefrontal and lateral temporal cortices, where increased FC were resulted; their results supported our hypothesis that high Aβ deposition related to attenuated FC, while medium level of Aβ deposition promotes FC as a compensatory response in CEN.

Salience network is a cingulate-frontal operculum system anchored in the dorsal anterior cingulate cortex and frontal insular cortex; it is involved in detection, integration and filtering information related to interception and emotions [48]. Furthermore, it involved in attentional capture and dynamic cognitive control [49]. Neurodegenerative diseases, most notably AD and MCI involve to an increase in social-motional sensitivity relates to intensification of salience network functional connectivity. Later work revealed that SN has a key function to perceive and response to homeostatic demands to guide behavior [50]. Recent studies suggested that SN functions across both high-level cognitive and attention networks, involved in the regulation of DMN and CEN between state switching of the brain [51,52], it demonstrated a top down regulation on the DMN and CEN [53]. In particular, the anterior insular to initiate network switching leading to engaging and disengaging of DMN and CEN. The SN act as a hub to generate appropriate behavior responses to salient stimuli. Thus, dysfunction of SN can contribute to affective and cognitive dysfunctions [49]. Recent studies suggested that appropriate level of SN activity is essential to maintain an alerting signal for brain response initiation to salient stimuli [54]. Our study showed that both high and low Aβ groups showed increased IFC in SN, with positive correlation between IFC and Aβ accumulation. The hyperactivity of the SN may be based on the AD pathology, by recruiting other brain resources to maintain its capability in switching between DMN and CEN for attending to internal and external salient stimuli [46]. Further study is recommended to investigate the inter-network FC within the triple network and confirm the SN regulation capability.

Other than the triple functional connectivity networks, researchers focused in SRN recently for its effect on cognition association with neurodegenerative diseases. Aberrant functional connectivity in SRN was found not only in AD, but also in early stage MCI patients. For MCI patients, SRN increased functional connectivity within the network, whereas functional connectivity decreased in AD patients. Previous study proved that the functional damage was found in both SN and SRN [55,56,57], but the degree of impairment is severe in SRN and less severe in SN [55], suggested that compensatory patterns was found in SN only. Moreover, damage in SRN was as serious as in DMN, which the aberrant functional connectivity has been suggested to be related to AD pathology, starting from early stage of the disease with increased FC and progress to AD with reduced FC [58,59].

Our finding demonstrated that IFC in SMN was aberrant in both Hi and Low amyloid groups. The SMN composed of auditory, visual and motor cortices, plays an important role in receiving signals from external, determine the reaction and responds to it accordingly. The presence of Aβ deposition in drusen, extracellular space beneath the retinal pigment epithelium, or within the head of optic nerve in early stage AD patients [60]. Furthermore, they often demonstrated Aβ deposition in primary sensory and motor regions [61] and their performance is poor in the stage of MCI and getting worse in AD [62,63]. Our study demonstrated that IFC in SMN increased in the LowAmy group, suggest that activation of IFC to minimize the effect of amyloid deposition; while the IFC reduced as a result of severe amyloid load.

## 9. Limitation of Study

This study was conducted in a real-life clinical setting and the participant recruitment was consecutive, while the sample was limited. [^18^F]flutemetamol PET/CT scan was offered to diseased groups only, healthy controls did not undergo PET scan due to radiation consideration. The final diagnosis of each participant was not confirmed by histopathology as gold standard. Furthermore, the tau PET imaging, vascular comorbidity, white matter hyper-intensity were not included in this study; these techniques were commonly used for characterizing AD and its phenotypes were considered as a substitute of histopathology recently, and might be more commonly used in the future [64,65]. Furthermore, APOEƐ4 status of the subjects was not recorded, which was closely related to the dysregulation of Aβ deposition [66].

## 10. Conclusions

The level of amyloid-β accumulated demonstrated differential effects on IFC in different brain networks. As the treatment to reduce amyloid-β plaques deposition is available in the market, it may be an option for HiAmy group to improve their IFC in major brain networks.

## Figures and Tables

**Table 1:** Demographics of participants.

	HiAmy	LowAmy	HC	*p*-Value
Number of participants	27 (16 AD, 11 AmyMCI)	31 (13 VD, 18 NamyMCI)	25	
Age (mean ± SD)	74 ± 7.17 (55–87)	77 ± 6.23 (66–88)	72 ± 6.34 (60–85)	0.245
Sex (M:F)	16:11	11:20	16:9	0.412
HK-MoCA score	19 ± 5.91 (3–24)	20 ± 4.23 (7–27)	N/A	0.063

Between-group differences in age, sex and clinical performances were tested using the Kruskal–Wallis test according to the level of measurement. Statistical significance was set statistical significance was set at *p* < 0.05. *p*-Value quoted are the asymptomatic significance of the Kruskal–Wallis test. HiAmy: High amyloid group; LowAmy: Low amyloid group; HC: Healthy controlsHiAmy: High amyloid group; LowAmy: Low amyloid group; HC: Healthy controls.

## Data Availability

The clinical data and MRI images are not publicly available for patient privacy protection purposes.
